# Supplementary material for: Impact of Co-Occurrence of Obesity and SARS-CoV-2 Infection during Pregnancy on Placental Pathologies and Adverse Birth Outcomes: A Systematic Review and Narrative Synthesis
Source: Pathogens. 2023 Mar 27;12(4):524. doi: 10.3390/pathogens12040524 (PMC10140965; doi:10.3390/pathogens12040524)
Supplement: Supplementary file 1 [file pathogens-12-00524-s001.zip › viral infection obesity placenta supplemetary figures Pathogens FINAL.pdf]

Supplementary Table S1: Included studies in review of maternal SARS-CoV-2 and elevated BMI pregnancies

Author	Publication Year	Tittle	Article type	Viral infection
Ritchmann	2020	Fetal deaths in pregnancies with SARS-CoV-2 infection in Brazil: A case series	Case series	SARS-CoV-2
Schwartz	2020	Chronic Histiocytic Intervillositis With Trophoblast Necrosis Is a Risk Factor Associated With Placental Infection From Coronavirus Disease 2019 (COVID-19)	Case series	SARS-CoV-2
Menter	2021	Placental Pathology Findings during and after SARS-CoV-2 Infection: Features of Villitis and Malperfusion	Case series	SARS-CoV-2
Fan	2021	No obviously adverse pregnancy complications and outcomes of the recovered pregnant women from COVID-19	Case series	SARS-CoV-2
Jang	2021	Pregnancy Outcome, Antibodies, and Placental Pathology in SARS-CoV-2 Infection during Early Pregnancy	Case series	SARS-CoV-2
Hanna	2021	Underestimation of SARS-CoV-2 infection in placental samples	Case series	SARS-CoV-2
Watkins	2021	Defining Severe Acute Respiratory Syndrome Coronavirus 2 (SARS-CoV-2) Placentitis	Case series	SARS-CoV-2
Adhikari	2020	Pregnancy Outcomes Among Women With and Without Severe Acute Respiratory Syndrome Coronavirus 2 Infection	Cohort	SARS-CoV-2
Lu-Culligan	2021	Maternal respiratory SARS-CoV-2 infection in pregnancy is associated with a robust inflammatory response at the maternal-fetal interface	Cohort	SARS-CoV-2
Patberg	2021	Coronavirus disease 2019 infection and placental histopathology in women delivering at term	Cohort	SARS-CoV-2
Brien	2021	Pandemic stress and SARS-CoV-2 infection are associated with pathological changes at the maternal-fetal interface	Cohort	SARS-CoV-2
Joseph	2021	Maternal Antibody Response, Neutralizing Potency, and Placental Antibody Transfer After Severe Acute Respiratory Syndrome Coronavirus 2 (SARS-CoV-2) Infection	Cohort	SARS-CoV-2
Rosembloom	2021	Coronavirus disease 2019 infection and hypertensive disorders of pregnancy	Cohort	SARS-CoV-2
Articles removed from the study				

Okunade	2019	Selenium deficiency and pregnancy outcome in pregnant women with HIV in Lagos, Nigeria	Cohort	HIV
Zash	2021	Maternal weight and birth outcomes among women on antiretroviral treatment from conception in a birth surveillance study in Botswana	Cohort	HIV
Hirudayakanth	2021	Mid-thigh circumference as an indicator of nutritional status to predict adverse pregnancy outcomes among HIV-infected and HIV-uninfected women in Malawi	Cohort	HIV

Supplementary Table S2: Maternal co-morbidities in maternal SARS-CoV-2 infection and overweight/obesity during pregnancy in each study included in this review. The comorbidities most frequently reported in the majority of the selected studies (n=13) were diabetes (8/13), a form of hypertension (chronic hypertension or pregnancy induced hypertension) (7/13) and gestational diabetes (6/13).

Study	Study type	Maternal comorbidities
Schwartz (2020)	Case series	Gestational diabetes<br>Diabetes<br>Recurrent bacterial infections<br>Idiopathic thrombocytopenia
Menter (2021)	Case series	Maternal age<br>Gestational diabetes<br>Smoking<br>Pollinosis
Fan (2021)	Case series	Gestational diabetes<br>Diabetes<br>Mild preeclampsia<br>Pregnancy induced hypertension
Watkins (2021)	Case series	Pregnancy induced hypertension
Adhikari (2020)	Cohort	Diabetes<br>Gestational diabetes<br>Chronic hypertension<br>Preeclampsia with severe features
Brien (2021)	Cohort – Canada	Pregnancy induced hypertension<br>Diabetes
Brien (2021)	Cohort – UK	Diabetes
Patberg (2021)	Cohort	Diabetes<br>Gestational diabetes<br>Preeclampsia
Lu-Cullingan (2021)	Cohort	Diabetes<br>Hypertension<br>Preeclampsia
Rosembloom (2021)	Cohort	Chronic hypertension<br>Gestational hypertension<br>Diabetes<br>Gestational diabetes<br>Hypertensive disorder of pregnancy<br>History of hypertensive disorder of pregnancy<br>Smoking<br>Current substance abuse<br>Preeclampsia with and without severe features

Joseph (2021)	Cohort	Asthma (other pulmonary conditions)<br>Chronic hypertension<br>Diabetes<br>HIV & mental health
Ritchmann (2020)	Case series	Not applicable
Jang (2021)	Case series	Not applicable
Hanna (2021)	Case series	Not applicable

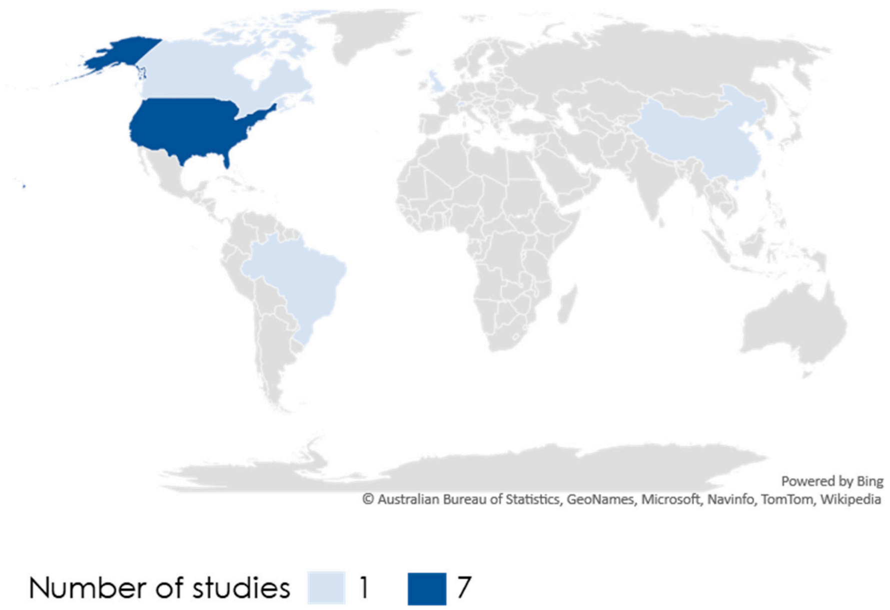

Supplementary Figure S1: World heatmap illustrating geographical location of each study included in the review. Data are shown for 13 studies from at least different 7 countries (one of the studies, Schwartz. *et al*, included patients from 5 different countries however, locations were not mentioned). Increasing study counts are represented by darker colours. 53% of the studies (n=7) were located in the United Stated of America. And the other 50% (n=6) were located in Canada, United Kingdom, Brazil, Switzerland, China, Korea.
